# Pandemic COVID‐19 as a challenge for telemedicine in the Czech Republic

**DOI:** 10.1002/hpm.3863

**Published:** 2024-11-04

**Authors:** Olga Angelovská, Karolína Dobiášová, Jolana Kopsa Těšinová

**Affiliations:** ^1^ First Faculty of Medicine Institute of Public Health and Medical Law Charles University Praha Czech Republic

**Keywords:** digitisation of health care, distant care, information and communication technologies, primary care, telemedicine

## Abstract

The COVID‐19 Pandemic contributed to accelerating the process of using information and communication technologies and digital technologies in healthcare management and delivery within healthcare systems. At that time, the Czech healthcare system faced the same problems as other European systems and struggled with a temporary limitation of direct provision of healthcare services. It was solved by switching to telemedicine. The Czech healthcare system used telemedicine to a minimal extent until then. Despite adopting the law on healthcare digitisation, it is still one of the countries with a lower level of digitisation of healthcare processes. The article presents the results of an exploratory expert investigation focused on the implementation and development of telemedicine in the Czech Republic. The conducted research aimed to identify problems related to the implementation of telemedicine in practice, place them in the broader framework of the healthcare system and structure them, propose possible solutions, and identify the future challenges of telemedicine in the Czech Republic. We based our study on the results of a three‐phase QUAL‐QUAN‐QUAL research. Data collection in the first phase took the form of individual semi‐structured interviews with patients (25) with practical experience in the field of telemedicine, followed by the second quantitative phase of the questionnaire survey with patients (650). The third qualitative phase included semi‐structured interviews with experts (17) with practical experience in telemedicine. The introduction and expansion of telemedicine require several fundamental changes. These include adjustments to the legislative environment and changes to the technological infrastructure, organisation of care and work. Several barriers have been identified at the healthcare system level, healthcare providers, healthcare professionals and patients.

## Highlights

- The research pointed to the prevailing satisfaction of patients with the care provided through the distance form using information and communication technology (ICT) during COVID‐19.
- Perceived barriers to the use of ICT on the part of patients are related to the fear of medical errors and information leakage, on the part of physicians to the uncertainty of the formal setting of telemedicine in the Czech system.
- Motivation to use ICT was to avoid infection, save time, motivate a healthier lifestyle and increase patient responsibility
- Successful implementation of telemedicine will require legislative changes, guidelines, the reorganisation of healthcare and educating doctors and patients to increase their digital literacy.

## 1 INTRODUCTION

As a result of its immediate negative impacts, the COVID‐19 pandemic has forced many temporary restrictions on direct service provision and affected social and health systems across Europe, regardless of their organisation and funding. The main cause of the changes in service delivery was the reduction of physical contact between providers and clients or patients. The weakening of traditional service delivery models has led to increased attention to the possibilities of digitisation. Digital solutions to care delivery have been visible, particularly in primary health care, where health systems have been pushed to maintain routine care during pandemics.^1^ Maintaining accessibility and ongoing management of health problems required changes in adopting or expanding information and communication technology (ICT) use during pandemics.^2^, ^3^ Thus, the use of telemedicine was a logical response to the risks associated with this emergency as well as the sudden increase in demand for health services.^4^ Telemedicine has become a way to maintain the absorptive capacity of primary care and thus maintain health system resilience in many countries.^5^

The use of telemedicine itself is a phenomenon that has been around for a while in healthcare. Influenced by many factors, such as the demographic ageing of the population or the continuous increase in healthcare spending, health policymakers have been striving to introduce new approaches in healthcare over the last 2 decades, with a focus on digital solutions. The all‐pervasive development and diffusion of ICT and digital technologies in healthcare management and delivery also contribute to this.^6^ The implementation of telemedicine, like the introduction of new technological solutions in other healthcare areas, has not only its enthusiastic supporters (optimists) who emphasise its possibilities and advantages. On the other hand, however, there are also opponents (pessimists) of introducing technology into healthcare who, on the contrary, stress the difficulties, possible obstacles and unintended consequences.^7^

There are many definitions and interpretations related to the term telemedicine. In general, telemedicine is defined as the use of information and communication technologies to deliver medical services remotely to provide solutions to problems of access, quality and cost of medical care.^8^ The World Health Organization^9^ defines telemedicine even more broadly as ‘The delivery of health care services, where distance is a critical factor, by all health care professionals using information and communication technologies for the exchange of valid information for diagnosis, treatment and prevention of disease and injuries, research and evaluation, and for the continuing education of health care providers, all in the interests of advancing the health of individuals and their communities’.

Some authors differentiate between telemedicine and telehealth, and telehealth should be a broader term that includes telemedicine but also includes acts performed by health professionals other than doctors, administrative appointments or telae‐education.^10^ However, the WHO report^9^ treats the two terms as synonymous. Telemedicine uses modern information and communication technologies to bridge the distance between health service providers and their clients, but also, for example, between doctors, enabling them to consult on their cases without the need to meet in person.

The basis of telemedicine is modern technology, through which it is possible to provide comprehensive patient care, from initial consultation and diagnosis through monitoring and treatment to prescribing medication or issuing electronic sick leave. All this is done through remote communication, remote data transfer and sharing of medical information in the form of audio, video, text or images.^9^ The transfer of information occurs using ICT. Telemedicine interventions, including remote health monitoring and telemedicine consultations, can contribute not only to improving the population's health status but also to their social and emotional well‐being, to improving access to health services, empowering patients and increasing their health literacy.^11^

Currently, four basic modalities of telemedicine can be distinguished, including synchronous videoconferencing, the asynchronous modality of remote patient monitoring, and mobile health (mHealth).^10^ The great potential of digital technologies lies in enhancing a patient‐centred approach, increasing the efficiency of work and operational processes, improving the security of health information, monitoring patients, personalising medicine, sharing information between different actors, as well as supporting decision‐making and management of people and processes in the health sector.^6^

The aim of the present article is to summarise the experience of patients and experts with the provision of health care by General practitioners (GPs) and outpatient specialists remotely using ICT to identify and objectify the possible barriers to access to this care and the risks in the context of its provision at an appropriate professional level (lege artis). The aim is also to identify and objectify the problem areas of distance communication in the outpatient healthcare segment, both during the COVID‐19 epidemic (and similar crises) and in everyday life, considering legal specifics.

### 1.1 Telemedicine in the Czech Republic

Telemedicine is an area of eHealth that has seen significant development in the Czech Republic due to the COVID‐19 pandemic. While eHealth encompasses all services and information transfer via ICT related to healthcare, telemedicine is defined as the use of ICT to provide healthcare services remotely, with communication taking place between providers among themselves or between healthcare users remotely and providers of that care.^9^ This definition of telemedicine is also used as a basis for its setting (implementation) in the Czech Republic.

Regarding the overall level of digitisation, the Czech healthcare system is assessed rather negatively compared to other European countries.^12^ The low level of digitisation of the Czech healthcare system is illustrated, for example, by the low proportion of physicians, namely 10% in 2020, who keep medical records in fully electronic form, while 69% of them still combine electronic and paper records and 18% keep them exclusively in paper form.^13^

The COVID‐19 pandemic did lead to advances in digitisation, but with limited success.^14^ GPs and outpatient specialists in the Czech Republic have started to use telehealth on a large scale in their practices to communicate with patients through ICT. For example, between March 2020 and January 2021 alone, GPs reported 3.5 million telehealth procedures to health insurers.^15^ However, the enforced transition to a new (distance) mode of healthcare delivery using ICT has taken place without prior adaptation and implementation of digital tools for communication with their patients.^16^

Thus, in the Czech Republic, the legislative development of e‐health is only at the beginning, and despite the adoption of the Act on the Electronicisation of Healthcare,^17^ the Czech Republic is still among the countries with a lower level of digitisation of healthcare processes. Exceptions are partial tools already in place (e.g. e‐prescription).

It is the result of the problematic definition of telemedicine in the current legislation, where the present concept of health service provision, with exceptions (e.g. second opinion consultation service), assumes the personal (physical) presence of the patient in the health care facility or the physical presence of a health care professional in the patient's social environment.^18^

To some extent, consultations can be provided by remote access, but without being defined in more detail by law. Thus, this concept makes it significantly more difficult for healthcare to be provided by remote access via ICT, that is, without the simultaneous presence of the doctor and the patient in the same place. Providing healthcare only by a ‘virtual’ provider who would not have a healthcare facility appropriate to the type and form of healthcare provided under the Health Services Act is wholly excluded.

Although a number of telemedicine solutions have been in operation in the Czech Republic for several years (e.g. in the field of cardiology), the definition of this care is not yet directly supported by legislation. Thus, despite the adoption of the law on computerisation, the area of telecare remains without special regulation. Therefore, the legal regulation of telemedicine applies only to general rules regulating mainly the provision of health services^18^, ^19^ and personal data protection.^20^ The same rules apply to telemedicine as to the provision of health care in general, that is, it must be provided at the appropriate professional level (lege artis), that is, according to the rules of science and recognised medical procedures, respecting the individuality of the patient, taking into account specific conditions and objective possibilities. However, the recommended practices in the field of health care deal with telemedicine only sporadically.^21^, ^22^

There is no mention of telemedicine in health legislation yet, which creates legal uncertainty for health service providers regarding the possibility and conditions of provision. In July 2023, the government approved a draft amendment to the Health Services Act,^23^ in which a definition of telemedicine health services appears for the first time. If Parliament also approves the amendment, it will explicitly state that it is a legally permitted method of providing health services and will set out the framework conditions for its use.

## 2 METHODS AND DATA SOURCES

The study uses a mixed research design, which is appropriate for exploring complex processes within a health system and provides a more plastic picture of the reality under investigation.^24^ It combined qualitative and quantitative research approaches in a sequential QUAL‐QUAN‐QUAL design. The first qualitative phase took place between February and April 2021. Data were collected through semi‐structured interviews with patients based on a pre‐designed scenario with open‐ended questions. These patients received primary health care provided by GPs in a distant form using ICT between March 2020 and March 2021. The condition was that the patients needed to consult a physician for their health problems or their child's health problems. It was not just about prescribing medicines, making appointments for preventive examinations, vaccinations, etc. A total of 25 interviews were conducted. Informants were selected through purposive sampling to ensure that the sample was as diverse as possible in terms of age, gender, residence and education.^25^

The results of the qualitative research served as the basis for the second quantitative phase, which took the form of a questionnaire survey. Based on the results of the qualitative research, a questionnaire was developed which included the following areas: initial screening, technical resources used, problem contacts using ICT, assessment of the previous experience of communicating with a doctor (GPs or outpatient specialist) remotely using ICT, the impact of the COVID ‐ 19 pandemic on healthcare uptake, barriers, motivators and expectations of healthcare uptake remotely using ICT. The Academy of Patient Organizations and the newly formed National Association of Patient Organizations (NAPO) assisted with the questionnaire distribution. APO is a platform for the education of Czech patient organisations, and NAPO is an umbrella organisation of patient organisations focused on all types of diseases and disabilities. Both organisations bring together more than 100 patient organisations operating in the Czech Republic.^16^, ^26^ The questionnaire was sent electronically to members of patient organisations affiliated with these two associations.

A total of 620 respondents from 65 patient organisations completed the questionnaire. Of this total, 540 respondents needed healthcare between March 2020 and June 2021. Two‐thirds of respondents (347, e.i. 64%) addressed their health problems remotely using ICT. Of these, 167 (31%) respondents used the services of GPs at their last consultation, and 180 (33%) used the services of an outpatient specialist. The sample included respondents of all age groups, both genders and different educational backgrounds. The technical equipment of the respondents was also monitored.

The questionnaire also included four questions selected from the HLS 19, an international standardised questionnaire for measuring health literacy, which was used in the Health Literacy Survey of the Czech population on a representative sample of 1650 persons between 10 and 24 November 2020. The results of this survey provided by the Institute for Health Literacy were used to compare the health literacy of the respondents. Statistical analysis of the data was performed using IBM SPSS Statistics 27.

In the third phase, a qualitative expert investigation was conducted to identify problems related to the implementation of telemedicine in practice, to situate them in the broader framework of the healthcare system and to structure them and to identify the challenges of telemedicine in the Czech Republic in the future. Data were collected through individual semi‐structured interviews with experts with practical experience in telemedicine. The experts were selected through a purposive sampling method^25^ to represent different actors in the field of telemedicine implementation: representatives of patient organisations, physicians from different segments of healthcare (primary, outpatient specialised and hospital) and specialities (general medicine, paediatric and adolescent medicine, diabetes, surgery, cardiology and dermatology) and representatives of health insurance companies (see Table 1). The interviews were conducted according to a pre‐designed script based on a literature study. The interviews took place in the period May‐August 2021. Regarding the protective measures related to the COVID‐19 pandemic, most interviews were conducted online (via video), while some were conducted in person. In total, 17 experts took part in the research. Except for two cases where field notes were taken, all interviews were recorded and transcribed verbatim. Subsequently, transcripts of individual interviews (including field notes) were subjected to thematic analysis.^27^ Our analysis focused on findings related to primary care.

**TABLE 1:** The individual expert interviews.

Expert	Area of expert knowledge	Form of data collection
E 1	Patient organisation	Video interview
E 2	Patient organisation	Video interview
E 3	Patient organisation	Video interview
E 4	Patient organisation	Video interview
E 5	Patient organisation	Video interview
E 6	Patient organisation	Video interview
E 7	Patient organisation	Video interview
E 8	Patient organisation	Video interview
E 9	General practitioner for children and adolescents	Video interview
E 10	General practitioner	Video interview
E 11	General practitioner	Video interview
E 12	Outpatient specialist	Video interview
E 13	Outpatient specialist	Video interview
E 14	Outpatient specialist and hospital doctor	In person
E 15	Doctor in hospital	In person
E 16	Representative of a health insurance company	In person
E 17	Representative of a health insurance company	In person

A limitation of the research is that the quantitative and qualitative data collection took place at the time of COVID‐19, and therefore some of the health problems addressed were related to COVID‐19. The questionnaire survey did not differentiate whether the episodes were related to COVID‐19 or to the resolution of other health problems.

## 3 RESULTS

### 3.1 Experience with telemedicine during COVID‐19

Health literacy plays an important role in the use of health services.^28^ The Czech Republic participated in the Health Literacy Survey 2019 (HLS19), run by the Action Network on Measuring Population and Organizational Health Literacy.^29^ Data collection in the Czech Republic took place in 2020.^30^ When comparing selected health literacy indicators of a representative sample of the Czech population in HLS19 and respondents of our research (i.e., a sample of members of patient organisations), health literacy is comparable for both groups in selected indicators (e.g., orientation in the health care system). Representatives of patient organisations differ in having higher health literacy in terms of following a doctor's or pharmacist's recommendations. Compared to the general population, our sample, given that we surveyed members of patient organisations, included patients who have chronic illnesses and, as a result, greater experience with healthcare utilisation or patients who are more involved in health policy development and implementation.

Another essential prerequisite for ICT use is technical equipment. In our sample, 97% of respondents in our questionnaire survey have an Internet connection at home, and 90% have a computer at home. When communicating remotely with a doctor, the mobile phone is the predominant technical means of communication (about 90%). Half of the respondents (54%) use a computer as a second means. In addition, half of the respondents have installed mobile health applications and use freely available devices to monitor their health status. Concerning monitoring devices, 15% have a doctor‐issued and recommended monitoring device.

Apart from ePrescription (90%) and making an appointment with a doctor (76%), the most common ways of addressing health issues were via distance (86%) and sending test results or photos of a health problem (39%). In terms of how a health issue is addressed, phone calls (95%), email correspondence (59%) and text messages (30%) dominate. Communication via specialised health apps and password‐protected platforms and video calls were used minimally (5% and 3%, respectively).

In expert interviews, all the experts from patient organisations and doctors were positive about the introduction and use of e‐prescriptions.

The ePrescription is a great tool because before we had to write paper prescriptions and patients had to come in person to get them. It works really very well. (E11)

The prescriptions over the phone are very convenient for me because, in the past, I often had to wait a long time in the GP's waiting room just for a prescription. (E4)

They agreed that this tool has protected patients and doctors from infection during the COVID‐19 pandemic but also saved considerable time and resources.

### 3.2 Motivation to use ICT

Based on their experience in practice, all the experts, especially the physicians, agreed that the COVID‐19 pandemic in the Czech Republic has accelerated the use of telemedicine in practice. Also, the questionnaire research showed that the impact of a pandemic had significantly affected the motivation for distance care using ICT. Here, motives related to the pandemic (avoiding infection, not infecting others) prevailed. Saving time is another essential motivational factor. Respondents were significantly less likely to agree that health monitoring using technical means motivates healthier health styles (40%) and greater patient responsibility (43%). Similar benefits of using ICT have been confirmed by interviewed experts (e.g. saving time and travel costs for patients and protecting patients from infection).

Motivation to use ICT in healthcare is positively correlated with education. People are more motivated with higher educational attainment. Personal experience with using ICT in health care during the study period also played a positive role compared to those who only visited the doctor in person. Personal experience led to higher motivation.

Patients who used remote monitoring using a physician‐issued device compared to those who did not use monitoring devices were significantly more likely to believe that remote monitoring motivates healthier lifestyles and greater patient responsibility.

Concerning the motivation on the part of physicians to use telemedicine, experts cited the reimbursement of telemedicine procedures from public health insurance as an important motivating aspect for them. It is a significant motivator for their use on the part of patients too.

If insurance companies do not cover telemedicine, the motivation of doctors and patients will be minimal. (E7)

Younger patients with certain types of diabetes use glycaemic monitoring because their health insurance covers it. Older patients are not yet covered. That plays a significant role because patients don't get it with their own money. (E12)

Experts from health insurance companies confirmed the positive attitude of insurance companies towards the reimbursement of selected telemedicine procedures. However, at the same time, they stressed that the decisive factor would be the effectiveness of the funds spent.

For us, as payers of care, the goal is to increase the efficiency of the entire system and reduce costs with the help of telemedicine. (E 17)

### 3.3 Barriers to the use of ICT for care

Despite the mentioned benefits, experts also pointed to many barriers to the use of telemedicine, both on the part of patients and healthcare providers. These included a lack of information about the possibilities and ways of using telemedicine, a lack of competence in working with technology and a lack of computer literacy. The lack of access to modern technologies and technical facilities for some patient groups was also mentioned as a serious barrier.

The more advanced the technology, the more it only works with modern operating systems, and not everyone can afford to buy a new phone every two years. We've got an enormous income gap here… Or the patient can't download data because he doesn't have internet. (E2)

So I think a lack of information in general can be a blocker to the use of telemedicine, then age, and of course, some technological ineptitude of that patient. (E5)

Low computer literacy is a problem, especially at older ages. (E8)

On the part of doctors, a certain ‘reluctance’ to change the established system also proved to be a barrier to introducing new telemedicine services.

Certainly, the most significant barrier is a combination of the fact that a significant number of GPs are close to retirement, so they have specific ways of running a practice and do not want to change them… (E3)

I'm a little older now, so I'm having a more challenging time learning these things, and I'm not really in the mood for big advances. I'm glad when this works for me… (E9)

During the second phase of our research, in our questionnaire survey, most respondents experience minimal barriers concerning remote healthcare using ICT and other technical means. Only a relatively small proportion of respondents (ranging from 8% to 12%) perceive the availability of technical devices and their ability to operate them as a barrier to using technical devices in healthcare. However, when designing policies aimed at implementing telemedicine, it is necessary to take into account vulnerable groups that lack digital competencies, as mentioned by experts. Slightly more perceive fear of medical malpractice (23.7%) and fear of information leakage (21%) as barriers, yet more than one‐fifth of respondents do not share these concerns.

Perceptions of barriers correlate with age, with individual barriers being perceived to a greater extent by older respondents. Barriers are also perceived less by those with a high school or college education than those with primary education or vocational schools. Asimilar is true for respondents who use smartphones, compared to those who use push‐button devices and those who have a health app installed or use remote health monitoring. These groups perceive fewer barriers. The experience of using remote healthcare via ICT during the study period also plays a role in the perception of barriers, with respondents without this experience perceiving barriers more.

### 3.4 Risks of providing care using ICT

Experts share patients' concerns about improperly delivered care. The main risks are the lack of information from the patient, the impossibility of visual and tactile contact, and the lack of knowledge of the patient. Doctors also expressed their own experience that telemedicine cannot be applied in all cases and that there are no clear rules about who can provide telemedicine consultations, to whom and under what conditions.

If it is by distance, then there is a risk that the patient may not even communicate much information, and the doctor needs more information to treat properly. (E12)

Telemedicine is pretty demanding. Of course, you may miss a number of things. You can't tell over the phone if the patient is yellow or has low blood cells, which of course you can see from the door during that in‐person visit. (E11)

In addition, it turned out that the Czech healthcare system is not sufficiently prepared for the implementation of telemedicine. Both doctors and representatives of patient organisations expressed concern that there are no guidelines on what a telemedicine consultation should look like. They pointed out, for example, that in a telemedicine consultation, without a clear structure of questions and a predetermined outline of the interaction with the doctor or nurse, the patient may miss something important. In addition to uncertainty about the process and structure of the telemedicine consultation, patients and employees of health insurance companies, in particular, expressed uncertainty about what form the output of the telemedicine consultation should take.

There is a lack of any rules about what the examination by the doctor should look like and what the outcome of the examination should be. For example, in my opinion, it should be a mandatory part of the report what the patient and the doctor have agreed on, and what the patient should do. (E2)

All practitioners then pointed to the possibility of leakage of personal data.

The problem is the data protection, the possibility of somehow abusing it. (E4)

’Those information flows are insecure, and it's not set up in a safe way for that patient. (E1)

Patients send photos to their mobile phone or email. Of course, if they send it to me, it's their voluntary decision because it's not encrypted. (E9)

According to some experts, so far, only some health service providers use secure communication channels with patients.

We use a lot of email communication ‐ we don't use the password‐protected app yet. (E10)

This was confirmed by the patient questionnaire survey data, which showed that only 8% of the patients surveyed communicated with their doctor via specialised healthcare apps and password‐protected platforms.

### 3.5 Risks of using ICT and data sharing

In the case of the implementation and development of telemedicine, experts pointed out that various telemedicine devices, including various applications, are increasingly used in practice and bring a number of advantages in treatment. At the same time as technology's advantages, the experts expressed some uncertainty about which of these devices is safe, what can be used and under what circumstances.

There is no such thing as an app, but it really has to fit some criteria. It has to meet strict rules so that the doctor knows that the data he is getting is actually relevant and that he can work with it. (E1)

In connection with medical devices, they mentioned the difficulty of the approval process and its insufficient institutional and legislative anchoring in the Czech Republic. According to them, the public perceives the concept of telemedicine as unknown and unclear. Patients miss information about what telemedicine can offer them. Experts also agree that informing about the risks associated with using ICT safely should become an integral part of education.

Patients should learn what telecare is all about. For example, GPs should inform patients which services can be provided at a distance. (E6)

Primarily, the patient doesn't perceive what risks or abuses might come with it. Patients should be educated about the potential risks of telemedicine and how they should protect themselves. (E3).

Experts reflected on the possibility and ways of aggregating data from monitoring devices to save time for doctors and healthcare professionals and liability issues in using AI.

The questionnaire survey confirmed a significant willingness of patients to share information about their health status with doctors. Almost three‐quarters of respondents (74%) want their health information to be available to all doctors who treat them, and they also want online access to their health records (69%). Positive attitudes towards health data sharing were linked to education; the higher the respondents' education level, the more they were in favour of data sharing.

Experts interviewed also pointed out that a significant shortcoming of the current healthcare system is the lack of health data sharing, not only between patient and doctor but also between doctors and healthcare providers. Health data is also inaccessible to patients. While data sharing is already practised in some segments, it is only for individual elements of the computerisation of healthcare, not a whole and unified system. Barriers to data sharing include outdated systems currently in use and the unresolved issue of responsibility for patient data.

### 3.6 Time aspect and organisation of work

Some providers had already been using individual telemedicine services before the COVID‐19 pandemic. However, as a result of the pandemic, many of them changed their organisation of work, and telemedicine became an integral part of the health services provided. Concerning the introduction of telemedicine, many experts from the ranks of physicians and patients alike have pointed out that in the current functioning of the healthcare system, physicians often do not have enough time for telemedicine consultations. In particular, doctors mentioned that the huge increase in patients' interest in telemedicine from patients increases their workload.

Thanks to telemedicine, patients think they can reach a doctor anytime, anywhere. For example, on the weekend, on vacation, or at 10 p.m., they email or phone me pictures of their health problems. That's not possible either. (E 14)

Experts from health insurance companies also confirmed the increase in interest in telemedicine services.

At the moment, we are discussing, when looking at data from health insurers, the huge increase in telephone consultations with GPs. These are huge increases for some practices. (E16)

On the other hand, experts of patients' representatives mentioned that, especially for telemedicine consultations, doctors do not have a defined time, which leads to the fact that they provide them in parallel with the treatment of other patients in the form of attendance or patients can't get through to them. Also, in email communication, patients often wait several days or even a week for a response.

Most respondents in the questionnaire research rated their experience of ICT‐based care positively regarding the content and quality of communication. Over 80% of respondents were satisfied with this form of care. The time factor was rated lower, with 15% of respondents needing more space to ask questions and a quarter of respondents saying they were not given the same amount of time during the distance consultation as during the face‐to‐face visit, and just under half said that their doctor did not have a designated time in their working hours for distance consultations. This is related to the respondents' experience of not calling their doctor (75%), in most cases (62%), even repeatedly.

Some doctors reported that they already use the online ordering option. Representatives of patient organisations who had experience with this service were very appreciative of it and would expect it to be widely used in all healthcare facilities in the future.

In the future, it would be good if all patients could make an appointment with a GP online. So far only my gynaecologist has it, but it's great. (E4)

Within the questionnaire survey, about 80% of respondents thought that doctors should have an online booking system for patient appointments. Representatives of patient organisations (e.g. E3, E4) also stated in interviews that most doctors do not yet provide these services.

### 3.7 Expectation for future use of telemedicine

Expectations for future use of technology in healthcare were higher among respondents with higher educational attainment and those who use a healthcare application. In the future, respondents would most often like to use ICT and other technical means when consulting on test results (71%) and consulting on vaccinations (66%). More than half of the respondents would also use ICT and other technologies when dealing with an acute condition, consulting on the evolution of a health condition for chronic disease and remote health monitoring. They would be less likely to consult via ICT on lifestyle issues and obtain a second medical opinion. For education or training (e.g. how to use a medical device or how to rehabilitate), respondents would use ICT the least (37%), and two‐fifths (43%) would not use this option.

## 4 DISCUSSION

The COVID‐19 pandemic has highlighted the need for remote healthcare delivery using telemedicine tools.^31^ Telemedicine has proved its worth during the COVID‐19 years as a solution to the limitations of health service delivery.^32^ Administrative telemedicine tools such as e‐prescription, e‐disability or e‐quarantine have proved their worth. At the same time, there are still reserves for its further development. While clinical e‐consultation technology is gradually developing, large reserves remain in remote access to electronic medical records and the use of modern technology to communicate with physicians.^33^ The untapped potential of ICT is also illustrated by our research, which points to minimal use of video calls or specialised health apps and password‐protected platforms, as telecare was mainly delivered through phone calls, text messages and email communication. Foreign research has also shown lower use of video calls depending on age.^34^, ^35^

The research pointed to the prevailing satisfaction of patients with the care provided through the distance form using ICT, where patients appreciated the time saving and the use of health care even if they could not come to the doctor in person. International studies have reached similar conclusions.^11^, ^36^, ^37^ However, the main motive during the pandemic was protection against infection. Patients in the questionnaire survey and physicians in expert interviews emphasised the benefits of ICT, which helped them to reduce personal contacts and thus avoid infection or protect others from infection. Experts also mentioned the telemedicine reimbursement from public sources (like public health insurance) as a motivating factor. The barriers of cost and reimbursement are the most frequently mentioned organisational barriers in the literature.^38^, ^39^

Patients have reservations about the duration of the telemedicine consultation and the possibility to ask additional questions. On the contrary, interviewed physicians mentioned the heavy time burden and unclear time boundaries for providing care. Thus, successful implementation of telemedicine will require not only changes in the technological infrastructure, but also in the organisation of healthcare and the organisation of healthcare professionals.^40^

The current Czech legislation provides insufficient legal certainty for the provision of telemedicine services. The legal framework hindering the expansion of telemedicine has already been significantly liberalised in many countries.^41^, ^42^ While guidelines are being developed abroad for various fields of healthcare provided by distance mode^43^, ^44^, ^45^ our research has shown that in some medical specialities guidelines are not available in the Czech Republic.

Experts have repeatedly warned about the lack of digital literacy as a barrier to the use of telemedicine on the part of both doctors and patients. Age was a risk factor mentioned by the experts, with 43% of GPs in the Czech Republic being over 60 years of age.^46^ International researchers reflected on the risk of inaccessibility of telecare for vulnerable groups. These include, for example, the elderly, low‐income groups, people with low literacy or immigrants and ethnic minorities, that is, groups at risk of digital exclusion with limited access to digital technologies or lower digital literacy.^47^, ^48^, ^49^ The risk of inaccessibility of telecare for vulnerable groups, concretely elderlies, has also been pointed out by experts and patients in our survey results.

Experts in interviews and patients in the questionnaire survey expressed concerns about the correct care provided due to insufficient information or limitations resulting from distance contact. Similar concerns have been identified in other research.^50^ Nevertheless, our research revealed positive expectations from patients and experts regarding the use of telemedicine in the future, which is consistent with other research.^51^, ^52^

## 5 CONCLUSION

Real‐world experience with telemedicine has dramatically accelerated its adoption and integration into healthcare infrastructure worldwide. The healthcare industry and the new situation created pressure to accelerate the digitisation of the Czech healthcare system, but the Czech healthcare system was not ready to respond quickly. Therefore, developments in those areas were at least partially implemented in the system. Now, an opportunity is being created for their permanent integration into primary care. Overall, we are optimistic about the finding that experts and more than four‐fifths of the interviewed patients involved in patient organisations are motivated to use ICT in healthcare further. Their concerns about data security and their knowledge and technical barriers need to be taken into account for policymaking. Our research has shown several crucial issues for the future development of telemedicine. Firstly, a legal framework that precisely defines the boundaries of the use of telemedicine services and clarifies the relationships between doctors and patients in the context of telemedicine should be developed. In addition, medical societies need to develop detailed guidelines for the use of telemedicine in different disciplines, including primary care. Furthermore, secondly, there is a need to change the organisation of health service provision. Telemedicine education for healthcare professionals and support for patients in developing their digital competencies are also important.

## CONFLICT OF INTEREST STATEMENT

The authors declare no competing interests.

## ETHICS STATEMENT

Not applicable.

## Data Availability

The data that support the findings of this study are available on request from the corresponding author. The data are not publicly available due to privacy or ethical restrictions.

## References

[1] Silva CRDV , Lopes RH , De Goes Bay , et al. Digital health opportunities to improve primary health care in the context of COVID‐19: scoping review. In: JMIR human factors. Vol 9. (2) JMIR Publications; 2022. ISSN 2292‐9495. 10.2196/35380 PMC9159467 35319466

[2] Mann DM , Chen J , Chunara R , Testa PA , Nov O . COVID‐19 transforms health care through telemedicine: evidence from the field. J Am Med Inform Assoc. 2020;27(7):1132‐1135. 10.1093/jamia/ocaa072 32324855 PMC7188161

[3] Bokolo A . Investigating the implementation of telehealth and digital technologies during public health crisis: a qualitative review. Int J Health Plann Manag. 2023;38(5):1212‐1227. ISSN 0749‐6753. 10.1002/hpm.3681 37452470

[4] Garattini L , Badinella MM , Zanetti M . More room for telemedicine after COVID‐19: lessons for primary care? Eur J Health Econ. 2021;22(2):183‐186. 10.1007/s10198-020-01248-y 33231825 PMC7683583

[5] Burau V , Mejsner SB , Falkenbach M , et al. Post‐COVID health policy responses to healthcare workforce capacities: a comparative analysis of health system resilience in six European countries. Online. Health Pol. 2024;139:104962. ISSN 0168‐8510. 10.1016/j.healthpol.2023.104962 38104372

[6] Cannavacciuolo L , Capaldo G , Ponsiglione C . Digital innovation and organizational changes in the healthcare sector: multiple case studies of telemedicine project implementation. Technovation. 2022;120:102550. 10.1016/j.technovation.2022.102550

[7] Ziebland S , Hyde E , Powell J . Power, paradox and pessimism: on the unintended consequences of digital health technologies in primary care. Soc Sci Med. 2021;289:114419. 10.1016/j.socscimed.2021.114419 34619631

[8] Tanriverdi H , Iacono CS . Diffusion of telemedicine: a knowledge barrier perspective. Telemed J. 1999;5(3):223‐244. 10.1089/107830299311989 10908437

[9] WHO . Telemedicine. Opportunities and Developments in Member States. WHO; 2010. https://apps.who.int/iris/bitstream/handle/10665/44497/9789241564144_eng.pdf

[10] Frehse AE . Overview and history of telehealth. In: Telemedicine. (Vol 2021. pp. 3‐14) Springer International Publishing; 2021;ISBN 3030640493. ISSN 2197‐7372. 10.1007/978-3-030-64050-7_1

[11] Eze ND , Mateus C , Cravo Oliveira Hashiguchi T . Telemedicine in the OECD: an umbrella review of clinical and cost‐effectiveness, patient experience and implementation. PLoS One. 2020;15(8):e0237585. 10.1371/journal.pone.0237585 32790752 PMC7425977

[12] OECD/European Observatory on Health Systems and Policies . Česká Republika: Zdravotní Profil Země 2021. Vol 202. OECD Publishing; 2021. 10.1787/a3017bfb-cs

[13] Český statistický úřad . (2021) Informační technologie ve zdravotnictví v České republice. (Czech Statistical Office report from 15. 12. 2021).

[14] KPMG . Studie – Připravenost ČR na digitalizaci zdravotnictví. 2022. ATDZ. 126. https://www.atdz.cz/upload/news/ATDZ_KPMG_Studie.pdf

[15] Knížek T . Digitalizace zdravotnictví: Využití dat přinese více efektivity pro pacienty, lékaře i pojišťovny. Redakce dreport. 2022. https://www.dreport.cz/blog/digitalizace‐zdravotnictvi‐vyuziti‐dat‐prinese‐vice‐efektivity‐pro‐pacienty‐lekare‐i‐pojistovny/

[16] Těšinová J , Dobiášová K , Dušek Z , Tobiášová A . Development of telemedicine in the Czech Republic from patients’ and other key stakeholders’ perspective. Online. Front Public Health. 2023;11:1202182. ISSN 2296‐2565. 10.3389/fpubh.20231202182 37937075 PMC10626478

[17] Act No. 325/2021 Coll., on the electronicisation of health care.

[18] Act No. 372/2011 Coll., on health services and conditions of their provision, as amended.

[19] Act No. 89/2012 Coll., civil code, as amended.

[20] Regulation (EU) . No 2016/679 of the European Parliament and of the Council of 27 April 2016 on the protection of natural persons with regard to the processing of personal data and on the free movement of such data and repealing Directive 95/46/EC (General Data Protection Regulation) and Act No 110/2019 Coll., on the processing of personal data.

[21] Mucha C , Býma S , Šonka P , et al. Telemedicína: Doporučené diagnostické a terapeutické postupy pro všeobecné praktické lékaře [online]. Společnost všeobecného lékařství ČLS JEP. 2020;13. https://www.svl.cz/files/files/Doporucene‐postupy/2020/DP‐Telemedicina.pdf

[22] Dubanská B . Právní rámec telemedicíny jako způsobu poskytování zdravotní péče. Čas Lék čes. 2021;160:280‐281.

[23] Government bill amending act No 372/2011 Coll., on health services.https://odok.cz/portal/veklep/material/KORNCMACTGI2/

[24] Fetters M , Curry L , Creswell JW . Achieving integration in mixed methods designs‐principles and practices. Health Serv Res. 2013;48(6 Pt 2):2134‐2156. 10.1111/1475-6773.12117 24279835 PMC4097839

[25] Robinson OC . Sampling in interview‐based qualitative research: a theoretical and practical guide. Qual Res Psychol. 2013;11(1):25‐41. 10.1080/14780887.2013.801543

[26] Dobiášová K , Kotherová Z , Numerato D . Institutional reforms to strengthen patient and public involvement in the Czech Republic since 2014. Health Pol. 2021;125(5):582‐586. ISSN 0168‐8510. 10.1016/j.healthpol.2021.03.011 33814202

[27] Braun V , Clarke V . Using thematic analysis in psychology. Qual Res Psychol. 2006;3(2):77‐101. 10.1191/1478088706qp063oa

[28] Sorensen, K , Pelikan, JM , Röthlin, F , et al. (2015) Health literacy in Europe: comparative results of the European health literacy survey (HLS‐EU). The European Journal of Public Health, Oxford University Press, 25(6), 1053‐1058, ISSN 1101‐1262. 10.1093/eurpub/ckv043 25843827 PMC4668324

[29] International Report on the Methodology, Results, and Recommendations of the European Health Literacy Population Survey 2019–2021 (HLS19) of M‐POHL. Austrian National Public Health Institute, 2021. www.uzg.cz/wp‐content/uploads/2022/01/HLS19_International‐Report‐002_0.pdf

[30] Kučera Z , Svačina Š , Šteflová A . Proměny úrovně zdravotní gramotnosti v Česku mezi lety 2015 a 2020. J Czech Physicians / Cas Lek Cesk. 2023;162(2/3):84. 37474291

[31] Bokolo AJ . Application of telemedicine and eHealth technology for clinical services in response to COVID‐19 pandemic. Health Technol. 2021;11(2):359‐366. ISSN 2190‐7188. 10.1007/s12553-020-00516-4 PMC7808733 33469474

[32] Okereafor K , Adebola O , Djehaiche R . Exploring the potentials of telemedicine and other non‐contact electronic health technologies in controlling the spread of the novel coronavirus disease (COVID‐19). IJITE. 2020;8(4):1‐13.

[33] Seifert B , Bezdíčková L , Mucha C , et al. Pandemie infekce COVID‐19 a primární péče. Společnost všeobecného lékařství ČLS JEP. 2020:21. [online]. https://www.svl.cz/files/files/Doporucene‐postupy/2020/DP‐Pandemie‐2020‐12‐02.pdf

[34] Chen J , Li KY , Andino J , et al. Predictors of audio‐only versus video telehealth visits during the COVID‐19 pandemic. J Gen Intern Med: JGIM. 2022;37(5):1138‐1144. ISSN 0884‐8734. 10.1007/s11606-021-07172-y 34791589 PMC8597874

[35] Bhatia R , Gilliam E , Aliberti G , et al. Older adults' perspectives on primary care telemedicine during the COVID‐19 pandemic. J Am Geriatr Soc. 2022;70(12):3480‐3492. 10.1111/jgs.18035 36169152 PMC9538237

[36] Gabrielsson‐Järhult F , Kjellström S , Josefsson KA . Telemedicine consultations with physicians in Swedish primary care: a mixed methods study of users' experiences and care patterns. Scandinavian journal of primary health care. 2021;39(2):204‐213. ISSN 0281‐3432. 10.1080/02813432.2021.1913904 33974502 PMC8293950

[37] Farr M , Banks J , Edwards HB , et al. Implementing online consultations in primary care: a mixed‐method evaluation extending normalisation process theory through service co‐production. BMJ Open. 2018;8(3):e019966. ISSN 2044‐6055. 10.1136/bmjopen-2017-019966 PMC5875620 29555817

[38] Scott Kruse C , Karem P , Shifflett K , Vegi L , Ravi K , Brooks M . Evaluating barriers to adopting telemedicine worldwide: a systematic review. J Telemed Telecare. 2018;24(1):4‐12. 10.1177/1357633x16674087 29320966 PMC5768250

[39] Giani E , Dovc K , Dos Santos TJ , et al. Telemedicine and COVID‐19 pandemic: the perfect storm to mark a change in diabetes care. Results from a world‐wide cross‐sectional web‐based survey. Pediatr Diabetes. 2021;22(8):1115‐1119. ISSN 1399‐543X. 10.1111/pedi.13272 34741569 PMC8661953

[40] Richardson E , et al. Keeping what works: remote consultations during the COVID‐19 pandemic. Eurohealth. Eurohealth, 26(‎2)‎:73‐76. 2020 WHO. Regional Office for Europe.

[41] Kichloo A , Albosta M , Dettloff K , et al. Telemedicine, the current COVID‐19 pandemic and the future: a narrative review and perspectives moving forward in the USA. Fam Med Community Health. 2020;8(3):e000530. 10.1136/fmch-2020-000530 32816942 PMC7437610

[42] de Oliveira AA , Soares AB , de Andrade Palis A , et al. On the use of telemedicine in the context of COVID‐19: legal aspects and a systematic review of technology. Res Biomed Eng. 2022;38(1):209‐227. 10.1007/s42600-021-00133-8

[43] Hall TL , Connelly L , Staton EW , et al. Points of concordance, points of discordance: a qualitative examination of telemedicine implementation. J Am Board Fam Med. 2022;35(3):517‐526. 10.3122/jabfm.2022.03.210325 35641043

[44] Venkatesh SK , Welle CL , Miller FH , et al. Reporting standards for primary sclerosing cholangitis using MRI and MR cholangiopancreatography: guidelines from MR working group of the international primary sclerosing cholangitis study group. Eur Radiol. 2022;32(2):923‐937. ISSN 0938‐7994. 10.1007/s00330-021-08147-7 34363134

[45] Intan Sabrina M , Defi IR . Telemedicine guidelines in south east Asia‐A scoping review. Online. Frontiers in neurology. 2021;11:581649. ISSN 1664‐2295. 10.3389/fneur.2020.581649 PMC7838484 33519669

[46] Šídlo L , Bělobrádek J , Maláková K . Všeobecní praktičtí lékaři v Česku: vývojové trendy a regionální rozdíly. Geografie. 2021;126(2):169‐194. 10.37040/geografie2021126020169

[47] Ryskina KL , Shultz K , Zhou Y , Lautenbach G , Brown RT . Older adults' access to primary care: gender, racial, and ethnic disparities in telemedicine. J Am Geriatr Soc. 2021;69(10):2732‐2740. 10.1111/jgs.17354 34224577 PMC8497419

[48] Qian AS , Schiaffino MK , Nalawade V , et al. Disparities in telemedicine during COVID‐19. Cancer Med. 2022;11(4):1192‐1201. ISSN 2045‐7634. 10.1002/cam4.4518 34989148 PMC8855911

[49] Duan GY , Ruiz De Luzuriaga AM , Schroedl LM , Rosenblatt AE . Disparities in telemedicine use during the COVID‐19 pandemic among pediatric dermatology patients. Online. Pediatr Dermatol. 2022;39(4):520‐527. ISSN 0736‐8046. 10.1111/pde.14982 35302248 PMC9115291

[50] Almathami HKY , Win KT , Vlahu‐Gjorgievska E . Barriers and facilitators that influence telemedicine‐based, real‐time, online consultation at patients' homes: systematic literature review. J Med Internet Res. 2020;22(2):e16407. ISSN 1438‐8871. 10.2196/16407 32130131 PMC7059083

[51] Woodall T , Ramage M , Labruyere JT , Mclean W , Tak CR . Telemedicine services during COVID‐19: considerations for medically underserved populations. J Rural Health. 2021;37(1):231‐234. ISSN 0890‐765X. 10.1111/jrh.12466 32613657 PMC7364549

[52] Danila MI , Gavigan K , Rivera E , et al. Patient perceptions and preferences regarding telemedicine for autoimmune rheumatic diseases care during the COVID‐19 pandemic. Arthritis Care Res. 2022;74(2010):1049‐1057. ISSN 2151‐464X. 10.1002/acr.24860 PMC9011874 35040274

